# Profiling the endocrine-disrupting properties of triazines, triazoles, and short-chain PFAS

**DOI:** 10.1093/toxsci/kfae131

**Published:** 2024-10-04

**Authors:** Maxim P Carlier, Peter H Cenijn, Timur Baygildiev, Jenny Irwan, Sylvia E Escher, Majorie B M van Duursen, Timo Hamers

**Affiliations:** Amsterdam Institute for Life and Environment, Section Environment and Health, Vrije Universiteit Amsterdam, 1081 HV Amsterdam, The Netherlands; Amsterdam Institute for Life and Environment, Section Environment and Health, Vrije Universiteit Amsterdam, 1081 HV Amsterdam, The Netherlands; Amsterdam Institute for Life and Environment, Section Environment and Health, Vrije Universiteit Amsterdam, 1081 HV Amsterdam, The Netherlands; Fraunhofer Institute for Toxicology and Experimental Medicine, Chemical Safety and Toxicology, 30625 Hannover, Germany; Fraunhofer Institute for Toxicology and Experimental Medicine, Chemical Safety and Toxicology, 30625 Hannover, Germany; Amsterdam Institute for Life and Environment, Section Environment and Health, Vrije Universiteit Amsterdam, 1081 HV Amsterdam, The Netherlands; Amsterdam Institute for Life and Environment, Section Environment and Health, Vrije Universiteit Amsterdam, 1081 HV Amsterdam, The Netherlands

**Keywords:** endocrine disruption, triazoles, triazines, PFAS

## Abstract

Persistent, mobile, and toxic compounds released to the environment are likely to pollute drinking water sources due to their slow environmental degradation (persistency) and high water solubility (mobility). The aim of the present study was to create in vitro hazard profiles for 16 triazoles, 9 triazines, and 11 poly- and perfluoroalkyl substances (PFAS) based on their agonistic and antagonistic effects in estrogen receptor (ER), androgen receptor (AR), and thyroid hormone receptor (TR) reporter gene assays, their ability to bind human transthyretin (TTR), and their effects on steroidogenesis. The triazole fungicides tetraconazole, bitertanol, fenbuconazole, tebuconazole, cyproconazole, difenoconazole, propiconazole, paclobutrazol, and triadimenol had agonistic or antagonistic effects on the ER and AR. Difenoconazole, propiconazole, and triadimenol were also found to be TR antagonists. The triazine herbicide ametryn was an ER, AR, and TR antagonist. The same 9 triazole fungicides and the triazines atrazine, deethyl-atrazine, and ametryn affected the secretion of steroid hormones. Furthermore, PFAS compounds PFBS, PFHxS, PFHxA, PFOS, PFOA, and GenX and the triazoles bitertanol, difenoconazole, and 4-methyl benzotriazole were found to displace T4 from TTR. These results are in line with earlier in vitro and in vivo studies on the endocrine-disrupting properties of triazines, triazoles, and PFAS. The present study demonstrates that this battery of in vitro bioassays can be used to profile compounds from different classes based on their endocrine-disrupting properties as a first step to prioritize them for further research, emission reduction, environmental remediation, and regulatory purposes.

A daily water intake of 2 to 2.5 l is recommended for adults, which makes water the principal constituent of a healthy diet (European Food Safety Authority [EFSA] 2016). Given this high intake, it is important to gain knowledge on the toxicity of compounds that have a high probability of polluting drinking water (Baken et al. 2018). Many triazines, triazoles, and short-chain poly- and perfluoroalkyl substances (PFAS) are persistent and water-soluble, enabling them to accumulate and distribute over long distances in hydrological systems. Combined with their poor removal in drinking water treatment plants, this leads to a relatively high probability of human exposure to such compounds (Reemtsma et al. 2016).

Some triazines are used as a component of melamine-formaldehyde resins, which are primarily used as coatings for interior surfaces, furniture, and tableware (Wang et al. 2023). Due to its widespread use and its persistent and mobile properties, melamine is now detected in many environmental waters and even in bottled and tap water (Zhu and Kannan 2020; Kolkman et al. 2021). Other triazines such as ametryn and atrazine are used as herbicides as they bind with the D1 protein, which is essential to photosystem II (Hess 2000). Although the use of triazine herbicides was prohibited in the European Union in 2003 (Sass and Colangelo 2006), atrazine is still detected in European ground and surface water (Guillon et al. 2019). Triazoles include mostly agricultural fungicides, which inhibit cytochrome P450 (CYP) 51 enzymes and thereby prevent the transformation of lanosterol to ergosterol (Parker et al. 2011), an essential component of the fungal cell wall. Because of their large-scale use, triazole fungicides are among the most frequently detected pesticides in surface waters of agricultural areas in Europe, Asia, and the Americas (Zubrod et al. 2019). PFAS are used for their water and grease-repellent properties, and short-chain PFAS are particularly produced as substitutes for the highly toxic, phased-out perfluorooctanoic acid (PFOA) and perfluorooctanesulfonic acid (PFOS) (Buck et al. 2011). Although pollution of surface water with PFOA and PFOS is still widespread, short-chain PFAS are now detected in even higher concentrations (Neuwald et al. 2022). The widespread contamination of drinking water and its sources, especially with persistent, mobile, and toxic (PMT) substances from various compound classes, is of growing concern for human health. Therefore, strategies to prioritize PMT compounds may support effective decision-making to reduce exposure.

In European chemical safety assessment, endocrine-disrupting properties need to be assessed. The societal attention for endocrine disruption spurred in the 1990s, when the US EPA started screening environmental pollutants for interfering with estrogen, androgen, thyroid, and steroidogenesis (EATS) modalities (Marty et al. 2011). These 4 modalities were chosen because they were considered most vulnerable to disruption by xenobiotics and because their disruption might cause the biggest health effects (Marty et al. 2011). Disruption of the 2 sex hormones, either in their synthesis or in their receptor interaction, is associated with decreased fertility, developmental defects, altered lipid metabolism, and increased incidence of hormone-sensitive cancers (Yilmaz et al. 2020). The disruption of thyroid hormones in early life, for example in their receptor interaction or their distribution to target tissues, is associated with developmental defects of the central nervous system (Yilmaz et al. 2020). Despite the variety of associated health effects, the identification of endocrine-disrupting chemicals (EDCs) in regulatory test strategies remains challenging. This is partly due to the complexity of the endocrine system and the multiple potential modes of action a compound may have. The EATS modalities are also the focus of European regulatory chemical safety assessments. For this, the Organization for Economic Cooperation and Development (OECD) provides a conceptual framework with a tiered approach for the assessment of EDCs (Browne et al. 2020). In this framework, results from in vitro tests with endpoints covering the estrogen, androgen, and steroidogenesis modalities are used alongside in vivo tests in the assessment of EDCs (Browne et al. 2020).

The aim of this study was to create in vitro hazard profiles based on endocrine-disrupting properties of 9 triazines, 16 triazoles, and 11 (mostly short-chain) PFAS. For this, we investigated the following readouts: agonism and antagonism toward the estrogen receptor (ER), androgen receptor (AR), and thyroid hormone receptor (TR) using reporter gene assays, alterations in the production of steroid hormones using the H295R steroidogenesis assay, and displacement of thyroxine (T4) from human transthyretin (TTR) using the FITC-T4 TTR-binding assay. Assays on transactivation of the ER and AR and on steroidogenesis are described in OECD test guidelines (OECD 2011, 2021, 2023), showing their relevance in the detection of endocrine disruptive effects for regulatory decision-making. The other assays, i.e., TTR-binding and TR transactivation, have no OECD test guidelines but they are cited in the OECD Guidance Document for evaluating chemicals for endocrine disruption (OECD 2018). The outcomes of these assays were used to derive in vitro hazard profiles and an evaluation was made of their applicability as a first step in prioritization for the allocation of resources for further research or measures to reduce human exposure.

**Table 1.** Tested compounds and their supplier.

Name	Abbreviation	Cas number	Supplier	Class
Sodium trifluoromethanesulfonate	TFMSA	2926-30-9	Sigma-Aldrich	PFAS
Perfluorobutane sulfonic acid	PFBS	375-73-5	Sigma-Aldrich	PFAS
Perfluorohexane sulfonic acid	PFHxS	355-46-4	LGC standards	PFAS
Perfluorooctane sulfonic acid	PFOS	1763-23-1	ABCR	PFAS
Sodium trifluoroacetate	TFA	2923-18-4	Sigma-Aldrich	PFAS
Perfluoropropionic acid	PFPrA	422-64-0	Sigma-Aldrich	PFAS
Perfluorobutanoic acid	PFBA	375-22-4	ABCR	PFAS
Perfluorohexanoic acid	PFHxA	307-24-4	ABCR	PFAS
Perfluorooactanoic acid	PFOA	335-67-1	Sigma-Aldrich	PFAS
Hexafluoropropylene oxide dimer acid	GenX	13252-13-6	ABCR	PFAS
Perfluoroethanesulfonic acid	PFEtS	2837-92-5	Kanto Chemical	PFAS
Cyromazine		66215-27-8	Sigma-Aldrich	Triazine
Atrazine		1912-24-9	Sigma-Aldrich	Triazine
Ametryn		834-12-8	Sigma-Aldrich	Triazine
Benzoguanamine		91-76-9	Sigma-Aldrich	Triazine
Melamine		108-78-1	Sigma-Aldrich	Triazine
Cyanuric acid		108-80-5	Sigma-Aldrich	Triazine
Deethyl-atrazine		6190-65-4	Sigma-Aldrich	Triazine
Ammeline		645-92-1	Sigma-Aldrich	Triazine
Ammelide		645-93-2	Sigma-Aldrich	Triazine
Tetraconazole		112281-77-3	Sigma-Aldrich	Triazole
Bitertanol		55179-31-2	Sigma-Aldrich	Triazole
Fenbuconazole		114369-43-6	Sigma-Aldrich	Triazole
Tebuconazole		107534-96-3	Sigma-Aldrich	Triazole
Difenoconazole		119446-68-3	Sigma-Aldrich	Triazole
Paclobutrazol		76738-62-0	Sigma-Aldrich	Triazole
Triadimenol		55219-65-3	Sigma-Aldrich	Triazole
Cyproconazole		94361-06-5	Sigma-Aldrich	Triazole
Propiconazole		60207-90-1	Sigma-Aldrich	Triazole
Pyroxsulam		422556-08-9	LGC standards	Triazole
Benzotriazole		95-14-7	Sigma-Aldrich	Triazole
4-Methylbenzotriazole		29878-31-7	Sigma-Aldrich	Triazole
5-Methylbenzotriazole		136-85-6	Sigma-Aldrich	Triazole
1,2,4-Triazole		288-88-0	Sigma-Aldrich	Triazole
Triazole acetic acid		28711-29-7	Sigma-Aldrich	Triazole
Triazole alanine		114419-45-3	ABCR	Triazole

**Table 2.** Cutoff values for in vitro hazard profiling.

	No response	Weak response	Strong response
Receptor activity assays	No response	EC10 or IC10 > 5 μM	EC10 or IC10 < 5 μM
TTR binding	No response	EC10 or IC10 > 100 nM	EC10 or IC10 < 100 nM
H295R	No response	EC_IR1.5_ > 2 μM	EC_IR1.5_ < 2 μM

**Table 3.** EC10/IC10 values and EC50/IC50 values (μM) of compounds showing agonism or antagonism toward the ER.

	ER agonism				ER antagonism			
	EC10	95% CI of EC10	EC50	95% CI of EC50	IC10	95% CI of IC10	IC50	95% CI of IC50
E2	7.4 × 10^−7^	4.9 × 10^−7^, 1.1 × 10^−6^	3.7 × 10^−6^	2.9 × 10^−6^, 4.5 × 10^−6^				
ICI-182.780					6.0 × 10^−6^	4.9 × 10^−6^ to 7.4 × 10^−6^	2.8 × 10^−5^	2.5 × 10^−5^ to 3.0 × 10^−5^
Ametryn					30.9	24.8 to 38.2	108	99.5 to 119
Bitertanol					0.53	0.2 to 1.3	9.3	6.2 to 14.7
Fenbuconazole					6.9	5.5 to 8.6	24.0	21.9 to 26.4
Tebuconazole					16.6	12.1 to 23.2	45.4	37.8 to 54.8
Cyproconazole	26.4	11.6 to 46.9	227	140 to 650				
Difenoconazole					0.55	0.33 to 0.94	2.2	1.7 to 2.8
Propiconazole					9.6	6.0 to 15.5	36.0	28.5 to 45.7
Paclobutrazol					13.6	8.5 to 21.2	84.5	68.7 to 109
Triadimenol	3.4	0.65 to 12.6	128	56.8 to 880				

**Table 4.** IC10 and IC50 values (μM) of compounds showing antagonism toward the AR or TR.

	AR antagonism				TR antagonism			
	IC10	95% CI of IC10	IC50	95% CI of IC50	IC10	95% CI of IC10	IC50	95% CI of IC50
Flutamide	0.13	0.1 to 1.8	1.0	0.9 to 1.2				
NH3					5.2 × 10^−3^	3.1 × 10^−3^ to 8.9 × 10^−3^	5.6 × 10^−2^	4.4 × 10^−2^ to 7.1 × 10^−2^
Deethyl-atrazine	34.1	12.2 to 71.0	132	45.0 to 219				
Ametryn	10.4	5.0 to 19.9	93.9	57.7 to 130	1.9	1.1 to 3.3	28.9	22.5 to 37.8
Tetraconazole	0.6	0.3 to 1.2	4.2	2.9 to 5.4				
Bitertanol	2.2	1.5 to 3.3	11.5	9.5 to 13.6				
Fenbuconazole	3.5	2.1 to 5.8	12.5	9.5 to 15.6				
Tebuconazole	1.5	0.8 to 2.9	8.1	5.6 to 10.6				
Cyproconazole	11.8	6.8 to 20.8	48.5	34.4 to 62.6				
Difenoconazole	1.4	1.0 to 2.1	4.6	3.7 to 5.6	0.64	0.36 to 1.1	4.1	3.1 to 5.5
Propiconazole	5.4	2.1 to 14.4	17.0	5.6 to 28.4	6.1	4.3 to 8.5	20.1	17.2 to 23.6
Paclobutrazol	15.6	8.0 to 31.2	56.7	34.9 to 78.5				
Triadimenol	8.8	5.8 to 13.4	37.0	29.5 to 44.6	6.3	3.7 to 10.7	21.3	16.4 to 28.3

## Materials and methods

### Test compounds

Thirty-six representative compounds from 3 structural classes were selected, namely triazines, triazoles, and short-chain PFAS. The persistence of the tested compounds was determined previously (Arp and Hale 2022) by various QSAR methods using the following criteria: The compounds had calculated degradation half-lives either lower than 40 d in water, or lower than 120 d in soil (Arp and Hale 2022). From the 36 tested compounds, only pyroxsulam was shown to not be persistent, but it was still included as it is both a triazole and a PFAS. Transformation products were included such as deethyl-atrazine, ammelide, ammeline, triazole acetic acid, triazole alanine, and 1,2,4-triazole to determine if the transformation leads to a marked change in endocrine disruption-related toxicity. Test chemicals all had purity >95% and were purchased from different suppliers (Table 1).

### Stock solution preparation

For most compounds, 100 mM stock solutions were made in DMSO (Thermo Scientific). For reasons of solubility and stability, exceptions were made for deethyl-atrazine and genX (100 mM in methanol), ammelide (100 mM in 0.1 M sodium hydroxide), ammeline and triazole alanine (25 mM in 0.5 M hydrochloric acid), and pyroxsulam (50 mM in acetonitrile). Stock solutions were stored at −20 °C. From these stock solutions, half logarithmic serial dilutions were prepared in their respective solvents with concentration ranges 0.06, 0.2, 0,6, 2, 6, and 20 mM for the reporter gene assays and concentration ranges 0.03, 0.1, 0.3, 1.3, and 10 mM for the TTR-binding assay. For the H295R assays, half logarithmic dilution series differed between compounds, depending on their cytotoxic effect. The highest concentration corresponded to concentrations resulting in less than 10% cytotoxicity.

### Cell culture

AR-Ecoscreen-GR-knockout M1 cells were constructed in our own lab by Zwart et al. (2017), using the original AR-EcoScreen cells (Satoh et al. 2004). Both cell lines are available from the Japanese Collection of Research Bioresources (JCRB) Cell Bank. GH3.TRE cells were kindly provided by Prof. Tinka Murk from Wageningen University (Freitas et al. 2011). VM7LUC4E2 cells were kindly provided by the late Prof. Michael Denison from University of California, Davis (Rogers and Denison 2000; National Institute of Environmental Health Sciences 2016). H295R cells were purchased from LGC Standards (product no. ATCC-CRL-2128).

VM7Luc4E2 and AR-Ecoscreen-GR-KO M1 cells were cultured in DMEM/F12 medium with glutamax (Gibco 31331-028) supplemented with 10% fetal calf serum (FCS) and 1% penicillin/streptomycin. VM7Luc4E2 and AR-Ecoscreen-GR-KO M1 cells were cultured under selection pressure with G418 (200 μg/ml; Sigma-Aldrich) or zeocin (200 μg/ml; Sigma-Aldrich), respectively. GH3.TRE-Luc cells were cultured in DMEM/F12 medium (Gibco 31331-038) supplemented with 10% FCS and 1% penicillin/streptomycin.

Phenol-free, low glucose DMEM (Gibco 11880-028) supplemented with 5% charcoal/dextran stripped FCS was used as assay medium for the VM7Luc4E2 and AR-Ecoscreen-GR-KO M1 cells. The assay medium for the GH3.TRE-Luc cells was DMEM/F12 medium (Gibco 31331-038) supplemented with 1% insulin-transferrin-selenium (ITS) (Corning 354350).

H295R cells were cultured in DMEM/F12 medium (Gibco 11039-021) supplemented with 1% ITS, 1% nu serum (Corning 355100), and 1% penicillin/streptomycin, and the assays were performed in the same medium.

### Reporter gene assays

#### ER-Luc

VM7Luc4E2 cells were cultured on assay medium 1 wk before seeding. After washing the wells of a 96-well cell culture plate (Greiner Bio-One Cellstar 655180) with ethanol, cells (2×10^4^/well) were seeded in a 100-µl volume and incubated for 24 h at 37 °C with 5% CO_2_. The exposure medium was prepared by diluting the compounds dissolved in their respective solvent 100× in assay medium by the epMotion5075 automated liquid handler. For the antagonism assays, the exposure medium was spiked with 7.4 pM of estradiol (E2; Sigma-Aldrich) as a reference agonist. The exposure medium was added (100 µl) to the cell culture plate resulting in final concentrations of 0.3 to 100 μM of the test compounds, in combination with a final concentration of 3.7 pM of E2 in the antagonism assays. All 36 compounds were tested once in a full dilution series, and active compounds were tested again in a full dilution series in 2 repeat experiments, whereas inactive compounds were tested in the highest noncytotoxic concentration again in 2 repeat experiments to confirm they were inactive. Calibration curves for agonistic and antagonistic action were obtained by exposing cells to 0.15 to 150 pM of E2 and 0.005 to 15 nM of ICI-182.780 (Sigma-Aldrich), respectively. All exposures were performed for 24 h at 37 °C in an atmosphere of 5% CO_2_.

After 24 h of exposure, cell viability was assessed by measuring the metabolic reduction of resazurine into fluorescent resorufine. Ten microliters of 440 μM resazurine (Sigma-Aldrich) in PBS was added to the 200 μl of medium in each well to obtain a final concentration of 22 μM of resazurine. After 2 h of incubation at 37 °C, fluorescence was measured using 550/585 nm (excitation/emission) wavelengths. We also visually inspected the shape of the cells to determine cytotoxicity. If viability decreased by more than 10% according to the resazurine assay, or if the shape of cells was altered, the data from that exposure condition was excluded from the statistical analysis.

After medium removal, cells were lysed in 50 μl lysis buffer (25 mM TRIS; 2 mM dithiothreitol [DTT]; 2 mM cyclohexanediaminetetraacetic acid; 10% glycerol; and 1% Triton X-100) and shaken at 700 rpm for 20 min. Luciferase production was assessed by adding 100 μl of glowmix (20 mM trycin; 1.07 mM C_4_H_2_Mg_5_O_14_; 2.67 mM MgSO_4_; 0.1 mM EDTA; 33.3 mM DTT; 270 mM coenzyme A; 470 mM luciferin; and 530 mM ATP), followed by a luminescence measurement and subsequent quenching by addition of 100 μl of brilliant black solution (50 mg/l; Sigma-Aldrich) in a Varioskan Flash (ThermoFisher) plate reader.

#### AR-EcoScreen

AR-EcoScreen assays were performed according to Satoh et al. (2004) with some modifications. AR-Ecoscreen-GR-KO M1 cells (1×10^4^/well) were seeded in 96-well cell culture plates (100 µl) and incubated for 24 h at 37 °C with 5% CO_2_. The cell culture medium was discarded and replaced with 100 μl of fresh assay medium. Test solutions in the exposure medium were similarly prepared and added as in the ER-Luc assays, except that dihydrotestosterone (DHT; 60 pM final concentration in the well) was used as a reference agonist in the antagonism assays instead of E2. Calibration curves were obtained by exposing cells to 10 to 1,000 pM of DHT in the agonism assays and 0.01 to 30 μM of flutamide (SigmaAldrich) in the antagonism assays. Exposure conditions (24 h; 37 °C; 5% CO_2_), resazurine assay, and luciferase measurements were similar to described for the VM7Luc4E2 cells.

#### TR-Luc

The GH3.TRE assays were performed following the methods of Freitas et al.(Freitas et al. 2011) In short, cells (3×10^4^/well) were seeded in 100 µl assay medium in 96-well cell culture plates. Test solutions in the exposure medium were prepared as described for the ER-Luc assay, except that triiodothyronine (T3, SigmaAldrich, 110 pM final concentration in the well) was used as a reference agonist in the antagonism assays instead of E2. Exposure medium was added to the cells immediately after seeding. Calibration curves were obtained by exposing cells to 3 to 10 000 pM of T3 in the agonist assays and 3 to 3,000 nM of NH3 (MedchemExpress, CAS number 447415-26-1) in the antagonist assays. Exposure conditions, resazurine assay, and luciferase measurements were similar as described for the VM7Luc4E2 cells.

### Data analysis reporter gene assays

For each experiment, mean relative luminescence unit (RLU) values (*n* = 3) were calculated for each exposure condition in Excel (Microsoft, Redmond, WA, United States). The mean RLU of the vehicle controls was subtracted from all other means. The means were then normalized as percentages of the maximum RLU response obtained for the positive control in the agonism assays and for the negative control in the antagonism assays. Concentration–response curves were fitted in Graphpad Prism 8 (Graphpad Software, La Jolla, CA, United States) using the formula *Y* = *A* + (*D*−*A*)/(1 + (*x*/EC50)^hillslope) in which *A* and *D* represent the minimum and maximum response, respectively. Fitting was done on the average RLU percentages per exposure condition determined in 3 independent experiments (*N* = 3). In the present study we report EC10/IC10 values and EC50/IC50 values of the active compounds to represent their potency both in the lower region and in the middle of their respective concentration–response curves.

### TTR-binding assay

Full dilution series of the 36 compounds were first screened in duplicate 3 times using a simplified version of the TTR-binding assay based on Hamers et al. (2020). Compounds that showed a response were further tested in triplicate 3 times in a more elaborate version of the assay according to Hamers et al. (2020), with minor modifications. All incubations were performed at room temperature.

In the screening tests, 48 μl of Tris-HCl buffer (pH 8.0) was added to each well of black low-binding 96-well plates. Two microliters of each test compound dissolved in their respective solvent was added to each well and the plate was shaken at 600 rpm for 5 min. A dilution series of thyroxine (T4) was also added as a positive control. Fluorescence was measured at 487/528 nm (excitation/emission) wavelength to check for autofluorescence of the compounds. About 100 μl of an in-house synthesized conjugate of T4 and fluorescein 5-isothiocyanate (FITC) dissolved in Tris-HCl buffer (220 nM) was added to each well and the plate was shaken in the dark at 600 rpm for 5 min. Fluorescence was measured again to check for possible autofluorescence or quenching by the test compound. Finally, 50 μl of human TTR solution (120 nM) in Tris-HCl buffer was added to each well and the plate was shaken in the dark at 400 rpm for 15 min. Fluorescence was measured for the third time to measure fluorescence enhancement for the TTR-bound FITC-T4 conjugate.

Fluorescence enhancement was determined for each well by subtracting its relative fluorescence units (RFU) before TTR addition from its RFUs after TTR addition. The resulting values were normalized to percentages of the vehicle control. A concentration–response curve was fitted through the data using the same model as in the reporter gene assays. A decrease in fluorescence indicates a decrease in FITC-T4 binding to TTR.

Compounds that were active in this assay were tested again in the original set-up (Hamers et al. 2020), where possible autofluorescence and quenching by the test compound were determined simultaneously with the fluorescence enhancement of the bound FITC-T4. Autofluorescence and quenching were determined (*n* = 3) by adding 2 µl of each test compound concentration dissolved in DMSO to 98 µl Tris-HCl buffer and 100 µl FITC-T4 solution (final concentration 110 nM). Fluorescence enhancement was determined (*n* = 3) by adding 2 µl of each test compound concentration to 48 µl Tris-HCl buffer, 100 µl FITC-T4 solution (final concentration 110 nM), and 50 µl TTR solution (final concentration 30 nM). After 15 min of incubation, fluorescence was measured and fluorescence enhancement was calculated by subtracting RFU without TTR from RFU with TTR. Further data analysis was similar to the screening assays.

### H295R assay

For the H295R assay, the culturing and exposure of cells were performed according to OECD Test Guideline No. 456 (OECD 2011). All experiments were performed using cells in passages 5 to 10. First, the highest noncytotoxic concentration (<10% decrease in viability) was determined for each compound with MTT assays. Next, the levels of 13 steroid hormones were determined in the medium after exposure of the H295R cells to the highest noncytotoxic concentration using LC-MS/MS. These concentrations were tested in triplicate (*n* = 3) in 3 independent experiments (*N* = 3), and all 9 samples were measured in LC-MS/MS. Finally, compounds that statistically significantly interfered (*P* < 0.05 in Dunnett post hoc test) with the production of at least one of the hormones were tested in half logarithmic dilution series. These dilution series were tested in 3 independent experiments (*N* = 3), with triplicate exposures in each of these experiments (*n* = 3). Supernatants of the triplicate exposures per experiment were pooled after the 48 h of exposure and the 3 pooled samples were measured with LC-MS/MS. This was done to keep the number of LC-MS/MS measurements manageable.

H295R cells (2×10^5^/well) were seeded in 24-well cell culture plates (Sarstedt 83.3922) and incubated in 1 ml volume for 24 h at 37 °C with 5% CO_2_. The exposure medium was prepared by diluting stock solutions 1,000× in the cell culture medium to obtain a final concentration of 0.1% of the solvent. Exposure was started by replacing cell culture medium with the exposure medium. This means the maximum final concentration of the compound was 25 μM for ammeline and triazole alanine, 50 μM for pyroxsulam, and 100 μM for all other compounds. On a separate quality control plate, cells were exposed to 10 μM forskolin (Sigma-Aldrich) and 1 μM prochloraz (Sigma-Aldrich) to serve as positive control for increased and decreased steroid hormone secretion, respectively. Exposure was started by replacing the culture medium in the cell culture plates with the exposure medium.

After 48 h of exposure, the supernatant medium from each well was transferred to the wells of a clean 48-well plate, and that plate was stored at −80 °C for later steroid measurement. MTT assays were performed by adding 500 μl of 0.5 mg/ml MTT (3-(4,5-dimethylthiazol-2-yl)-2,5-diphenyltetrazolium bromide; Sigma-Aldrich) solution in medium to each well of the 24-well cell culture plate and incubating for 1 h at 37 °C with 5% CO_2_. After incubation, the MTT solution was discarded and 500 μl of DMSO was added to each well to lyse the cells and dissolve the formazan crystals. Plates were shaken at 700 rpm for 10 min and absorbance was measured at 560 nm wavelength. Concentrations causing >10% decrease in cell viability were excluded from the hormone analysis, and test compounds were retested at a lower concentration.

Steroid hormone concentrations were measured using an LC-MS/MS method, as previously described (Evangelista et al. 2024). Hormones were concentrated from supernatants by solid phase extraction using Bond Elut Plexa cartridges, 30 mg sorbent, 1.8 ml (Agilent Technologies, Santa Clara, CA, United States). The cartridges were conditioned with 1 ml of 0.5% formic acid in methanol, and subsequent addition of 1 ml of 0.5% formic acid in water. A mix of 100 μl of H295R medium sample in 1 ml of 2% formic acid in water and 50 μl of isotopically labeled internal standards mix (Table S1) was loaded on the cartridges. These were then washed with 1 ml of water followed by 1 ml of water–methanol mixture (70:30, v/v). Cartridges were dried by vacuum and the hormones were eluted with 700 μl of methanol into 96-well-deep well polypropylene plates. Eluates were evaporated to dryness during approximately 2 h in a refrigerated CentriVap vacuum concentrator and reconstituted in 150 μl of water–methanol mixture (1:1, v/v). Additionally, a serial 12-point dilution series of a mix of native steroid hormones was prepared to fit a calibration curve in order to quantify steroid concentrations.

The reconstituted eluates and calibration standards were measured on a SCIEX Triple Quad 6500+ System with an electrospray ionization source (SCIEX, Framingham, MA, United States) using the settings described by Evangelista et al. (2024): curtain gas = 35 psi, temperature = 600 °C, ion source gas 1 = 70 psi, ion source gas 2 = 50 psi, ion spray voltage = 5,500 (positive mode), and −4,500 V (negative mode). The steroid hormones were separated prior to MS measurements on a Kinetex C18 LC Column (2.6 μm, 100 × 2.1 mm; Phenomenex, CA, United States) coupled to an Exion LC system (SCIEX, Framingham, MA, United States). The mobile phase consisted of 0.2 mM NH_4_F in water and pure methanol and was delivered at a flow rate of 0.6 ml/min in a gradient elution mode. The injection volume was 10 μl.

The estrogens estrone (E1) and 17β-estradiol (E2) were measured after derivatization of the eluates with dansyl chloride because the ionization efficiency of estrogens is low (Lin et al. 2007) and because they are secreted in low amounts by the H295R cells (Hecker et al. 2011). In short, the remaining 140 μl of the reconstituted SPE eluates in water–methanol mixture (1:1, v/v) and 6 dilutions of calibration standards were evaporated to dryness, and 40 μl of carbonate/bicarbonate buffer (pH 10.5) and 40 μl of dansyl chloride solution (1 mg/ml) in acetone were added. These dansylated samples were measured again with the same LC-MS/MS settings, except for some minor changes in the LC elution gradient which were described by Evangelista et al.(Evangelista et al. 2024).

The LC-MS/MS raw data were processed using AB Sciex OS Analyst version 1.6.2 (SCIEX, Framingham, MA, United States) to calculate fold changes by dividing the measured hormone concentrations by the hormone concentrations in the vehicle controls. The fold changes were used to fit a concentration–response curve in GraphPad Prism using the same model as in the reporter gene assays, and the EC_IR1.5_ was estimated for each compound. The EC_IR1.5_ corresponds to the concentration causing a 1.5-fold change in hormone production, i.e., 150% of vehicle controls for increased hormone levels or 66.7% for decreased hormone levels. The choice of 1.5-fold increase or decrease was based on the OECD guideline for the H295R steroidogenesis assay (OECD 2011), in which compounds that show more than 1.5-fold increase or decrease in at least 2 consecutive concentrations are categorized as active compounds in this assay. EC_IR1.5_ was preferred above EC50, because for some hormones no maximum or minimum level was reached within the concentration range tested, and no full sigmoidal concentration–response curve could be fitted. Consequently, no reliable EC50 value could be estimated. The EC_IR1.5_ is a more suitable metric for these data, as it uses a fixed response (i.e., 1.5-fold increase or decrease) in the lower region of the concentration–response curve (Escher et al. 2014).

Similarities and dissimilarities between different test compounds in their effect on steroid hormone profiles were further explored by hierarchical cluster analysis (Walters 1983) with Pearson correlation and by principal component analysis (PCA) (Walters 1983). For this purpose, the ^2^log-transformed fold changes of the different hormones at the highest noncytotoxic test concentration of each test compound were further analyzed in R using the pheatmap() and prcomp() functions, respectively.

### In vitro hazard profiling

In vitro hazard profiles of the 36 tested compounds were constructed based on all tested readouts. The compounds were categorized based on arbitrary cut-off values shown in Table 2 that were chosen to get the most contrast between the different compounds. In the H295R assay, the EC_IR1.5_ values for effects on progesterone, testosterone, E2, and cortisol levels were used because these hormones have the highest biological activity. A heatmap showing the in vitro hazard profiles was created in Graphpad Prism 8.

## Results

### Agonism and antagonism toward the ER

**Fig. 1.**
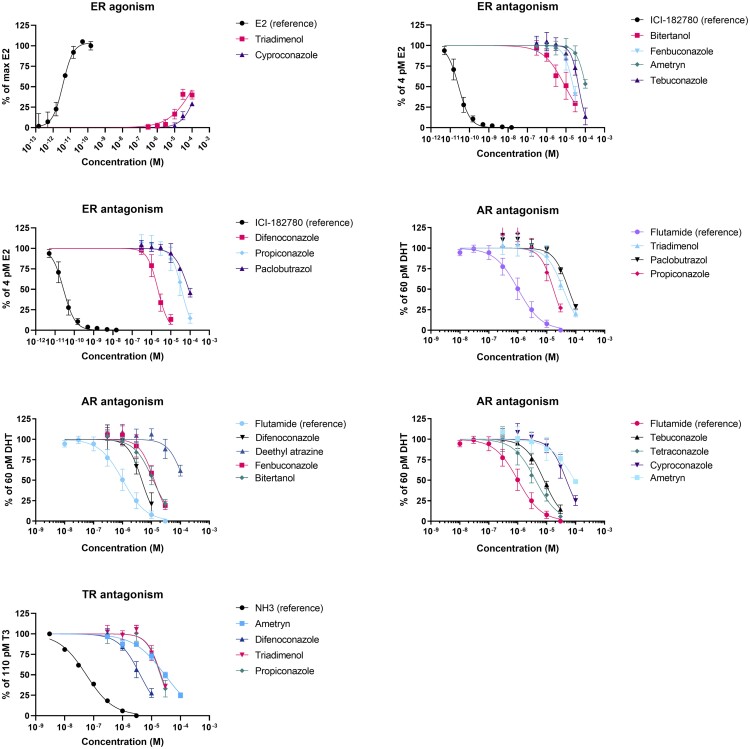
Concentration–response curves of compounds showing ER agonism or antagonism, AR antagonism, or TR antagonism. Only active compounds are shown. None of the other test compounds showed a response of at least 10% in the agonism and antagonism assays at any of the tested concentrations.

Exposures to 30 and 100 μM of difenoconazole and 100 μM of bitertanol and fenbuconazole resulted in a decrease in viability of more than 10%, and were therefore excluded from the concentration–response analysis. In the VM7Luc4E2 cell line, only triadimenol and cyproconazole showed weak agonism toward the ER (EC50 > 100 μM) compared with the reference compound E2 (EC50 = 3.7 pM; Table 3; Fig. 1). None of the other compounds showed a response at any of the 6 tested concentrations. On the other hand, 6 of the tested triazoles and 1 triazine showed antagonism toward the ER when coexposed with 3.7 pM E2. In the order difenoconazole < bitertanol < fenbuconazole < propiconazole < tebuconazole < paclobutrazol < ametryn, IC50 values increased from 2.2 to 108.0 μM (Table 3; Fig. 1). The test compounds were 5 to 7 orders of magnitude less potent than the ER antagonistic reference compound ICI 182780 (IC50 = 28 pM).

### Agonism and antagonism toward the AR

In the resazurine assay, significant cytotoxicity was measured after exposure to 30 and 100 μM difenoconazole and 100 μM of tetraconazole, tebuconazole, fenbuconazole, bitertanol, and propiconazole. These concentrations were therefore excluded from the concentration–response analysis. In the AR-Ecoscreen-GR-KO M1 cell line, none of the tested compounds had an agonist effect toward the AR at any of the 6 concentrations tested, whereas an EC50 of 60 pM was estimated for the reference compound DHT. Nine triazoles and 2 triazines showed antagonism toward the AR when coexposed with 60 pM DHT. In the order tetraconazole < difenoconazole < tebuconazole < bitertanol < fenbuconazole < propiconazole < triadimenol < cyproconazole < paclobutrazol < ametryn < deethyl-atrazine, IC50 values increased from 4.2 to 132.0 μM (Table 4; Fig. 1). For the antiandrogenic reference compound flutamide, an IC50 of 1.0 μM was observed, indicating that the compounds of interest were 4 to 132 times less potent AR antagonists than flutamide.

### Agonism and antagonism toward the TR

More than 10% cytotoxicity was measured in GH3.TRE cells after exposure to 30 and 100 μM of difenoconazole and fenbuconazole and after exposure to 100 μM of tetraconazole, tebuconazole, cyproconazole, bitertanol, triadimenol, and propiconazole. None of the compounds showed an agonistic effect on the TR at any of the tested concentrations, whereas the reference compound T3 showed an EC50 of 110 pM. After coexposure with 110 pM T3, the reference TR antagonist NH3 showed an IC50 of 56 nM. Antagonism toward the TR was observed in the order difenoconazole < propiconazole < triadimenol < ametryn, with IC50s increasing from 4.1 to 28.9 μM (Table 4; Fig. 1).

### TTR binding

**Fig. 2.**
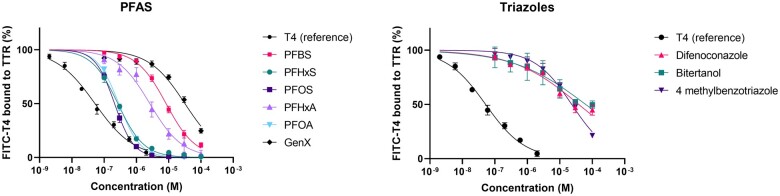
Concentration–response curves showing the FITC-T4 displacement from TTR by compounds that gave a response of at least 10% at any of the tested concentrations.

Results from experiments according to the initial set-up, i.e., simultaneous measurement of autofluorescence/quenching by the test compound and fluorescence enhancement of bound FITC-T4 (Fig. 2; Table 5) were very similar to results from the screening method, i.e., serial measurement of autofluorescence/quenching and fluorescence enhancement (Table S2 and Fig. S1). Nine test compounds were found to bind to TTR in the FITC-T4 displacement assay. In the order PFOS < PFHxS < PFOA < PFHxA < PFBS < 4-methylbenzotriazole < GenX < difenoconazole < bitertanol IC50 values increased from 0.21 to 62.7 μM (Table 5; Fig. 2).

**Table 5.** IC10 and IC50 values (μM) of compounds competing with FITC-T4 for binding to TTR.

	IC10	95% CI of IC10	IC50	95% CI of IC50
T4	2.4×10^−3^	1.8×10^−3^ to 3.3×10^−3^	5.2×10^−2^	4.6×10^−2^ to 5.9×10^−2^
Bitertanol	0.22	0.07 to 0.66	62.7	36.1 to 137
Difenoconazole	0.23	0.12 to 0.43	38.5	28.6 to 54.2
4-methylbenzotriazole	1.3	0.96 to 1.8	23.3	20.4 to 26.9
PFBS	0.82	0.68 to 0.99	8.3	7.6 to 9.0
PFHxS	3.4×10^−2^	2.8×10^−2^ to 4.2×10^−2^	0.27	0.25 to 0.29
PFOS	4.7×10^−2^	4.1×10^−2^ to 5.3×10^−2^	0.21	0.20 to 0.22
PFHxA	0.20	0.13 to 0.32	2.6	2.1 to 3.2
PFOA	5.3×10^−2^	4.6×10^−2^ to 6.1×10^−2^	0.28	0.27 to 0.30
GenX	1.9	1.3 to 2.9	31.9	26.8 to 38.4

### Effects on steroidogenesis

**Fig. 3.**
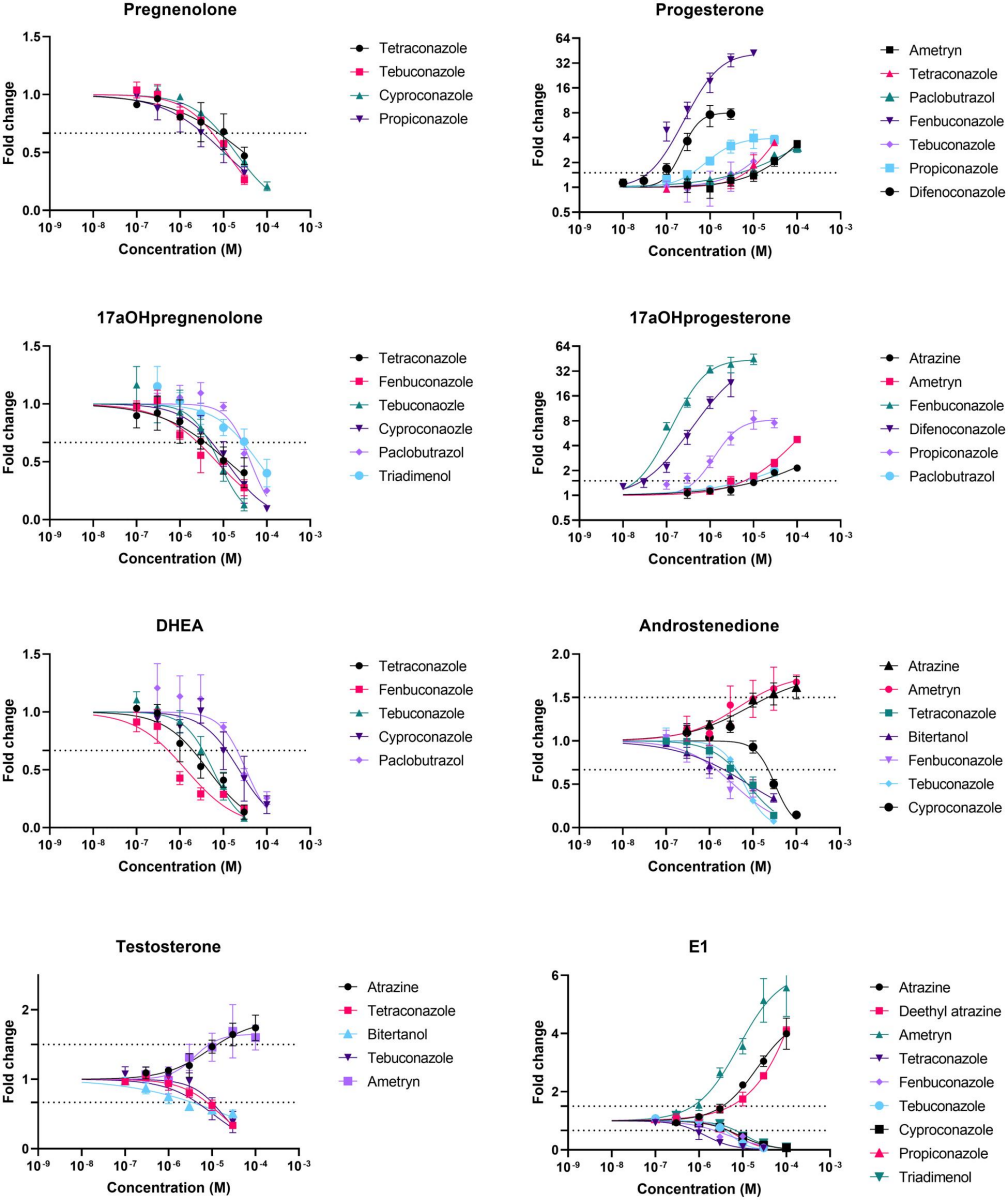

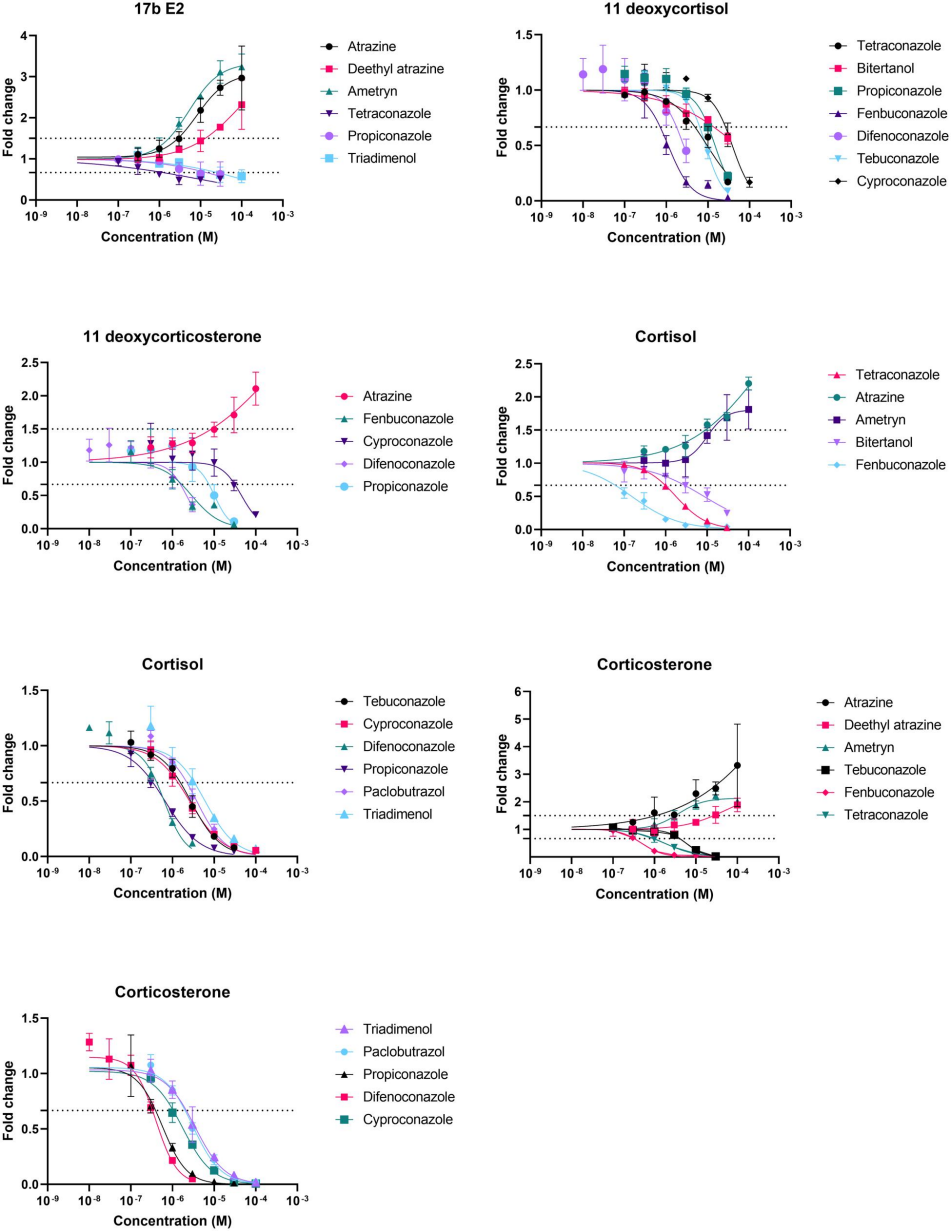
Concentration–response curves of compounds that increase or decrease levels of steroid hormones in the culture medium. The dotted line shows the 0.667- and 1.5-fold change in steroid hormone level compared with the vehicle-treated control, which is used to calculate the EC_IR1.5_. The *y* axis in the graphs on progesterone and 17a-OH-progesterone is in logarithmic scale to keep the 1.5-fold change discernable while showing the large fold changes induced by some compounds at the highest tested concentrations. DHEA, dehydroepiandrosterone; E1, estrone; E2, estradiol.

Effects on steroidogenesis were assessed by exposing H295R cells for 48 h to the test compounds. The highest exposure concentrations with <10% decrease in cell viability were found to be 3 μM for difenoconazole and 30 μM for tetraconazole, bitertanol, fenbuconazole, tebuconazole, and propiconazole. None of the other compounds showed more than 10% decreases in cell viability at their highest exposure concentrations of 25 μM for ammeline and triazole alanine, 50 μM of pyroxsulam, and 100 μM for all other compounds (data not shown). After exposure to noncytotoxic concentrations, 12 test compounds caused statistically significant changes in levels of 1 or more of 13 steroid hormones compared with vehicle-treated controls, i.e., atrazine, deethyl-atrazine, ametryn, tetraconazole, bitertanol, fenbuconazole, tebuconazole, cyproconazole, difenoconazole, propiconazole, paclobutrazol, and triadimenol. These compounds were further tested in dilution series, and the resulting concentration–response curves (Fig. 3) were used to calculate the EC_IR1.5_.

**Fig. 4.**
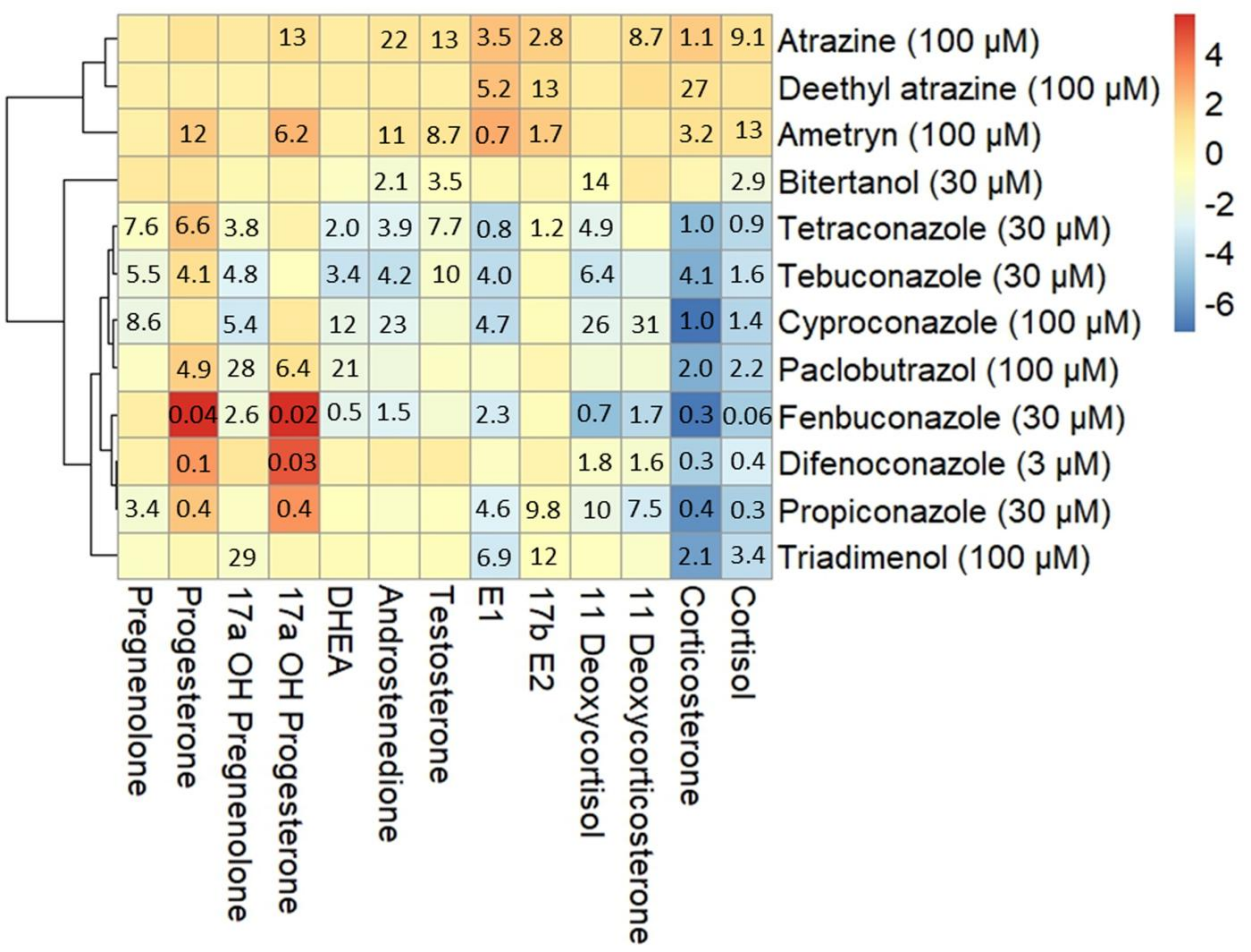
Clustered heatmap showing the effects of compounds affecting the levels of one or more steroid hormones in H295R media after 48 h, using Pearson’s correlation. The color shows the ^2^log transformed fold change at the highest, noncytotoxic exposure concentration (shown in parentheses) indicating a decreased (blue) or increased (red) hormone secretion by H295R cells. Values in the cells represent the EC_IR1.5_ value in μM indicating the exposure concentration at which the steroid levels change 0.667- or 1.5-fold compared with vehicle-treated controls. DHEA, dehydroepiandrosterone; E1, estrone; E2, estradiol.

A hierarchical clustering analysis of the ^2^log-transformed fold changes at the highest noncytotoxic exposure concentration resulted in a heatmap with 3 main clusters of compounds (Fig. 4). Cluster I consists of the triazines atrazine, deethyl-atrazine, and ametryn, which caused increased levels of progestogens, androgens, estrogens, and corticosteroids compared with vehicle-treated controls. Cluster II consists of tetraconazole, tebuconazole, cyproconazole, and paclobutrazol which showed increased levels of progesterone and decreased levels of androgens, estrogens, and corticosteroids compared with vehicle-treated controls. Cluster III consists of fenbuconazole, difenoconazole, and propiconazole, which are characterized by more than 8-fold increased levels of 17α-OH-progesterone compared with vehicle-treated controls. Within Cluster III, difenoconazole and propiconazole had no effect on androgen levels, but showed decreased levels of corticosteroids compared with vehicle-treated controls. Exposure to fenbuconazole resulted in decreased levels of androgens, estrogens, and corticosteroids compared with vehicle-treated controls. Bitertanol and triadimenol showed steroidogenic profiles that were distinctly different from the 3 clusters. Exposure to bitertanol resulted in decreased levels of androgens compared with vehicle-treated controls. Triadimenol led to decreased estrogen and corticosteroid levels compared with vehicle-treated controls. PCA showed similar results to the hierarchical clustering analysis (Fig. S2).

### In vitro hazard profiling

**Fig. 5.**
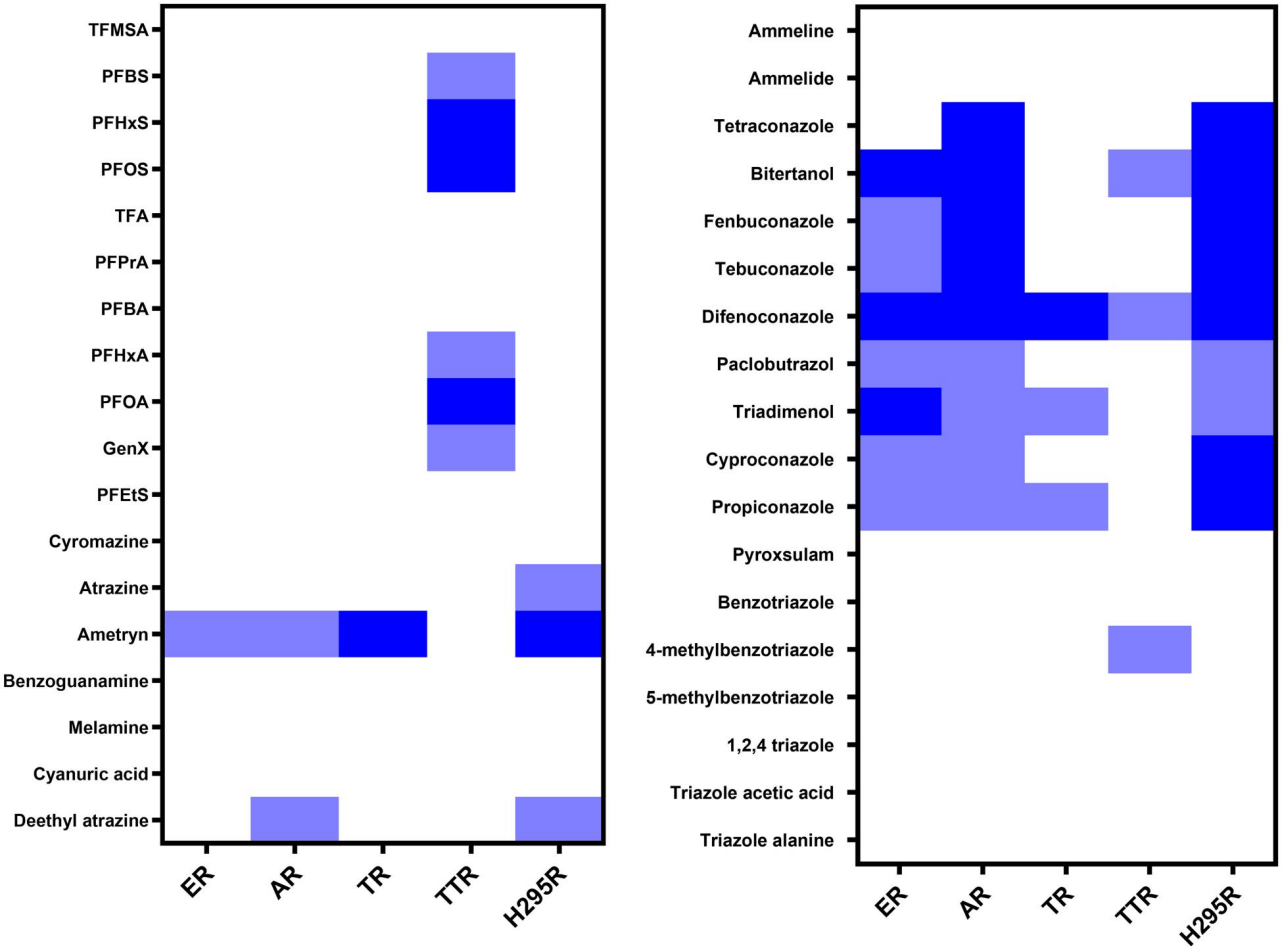
In vitro hazard profiles of the 36 tested compounds on all tested readouts. The column of ER, AR, and TR indicate both agonistic and antagonistic effects. The arbitrary cutoff values for the different categories in each readout are stated in Table 2. No response is shown in white, weak response is shown in light blue, and strong response is shown in dark blue.

In vitro hazard profiles were created for all 36 tested compounds based on the results of the receptor activity assays, H295R assay and TTR-binding assay (Fig. 5). The in vitro hazard profiles show that the tested PFAS only show activity in the TTR-binding assay. The triazole fungicides are characterized by activity in 3 readouts: ER, AR, and H295R. Finally, the readout TR does not seem to be informative as only 4 compounds showed activity and these compounds were also active in the ER, AR, and H295R assay.

## Discussion

The aim of the present study was to create in vitro hazard profiles based on the endocrine-disrupting properties of 36 persistent and mobile compounds and to use this information to identify the most hazardous compounds with respect to the EATS modalities.

### Estrogen and androgen receptor (ant)agonism

Our results on agonism/antagonism toward the hormone receptors show that triazole fungicides are mostly antiandrogenic and antiestrogenic. In fact, all tested triazole fungicides showed antagonism toward the AR, which is in line with earlier studies using reporter gene assays (Okubo et al. 2004; Kjærstad et al. 2010; Christen et al. 2014; Roelofs et al. 2014; Lv et al. 2017). The 7 nonfungicidal triazoles that were tested, including the final degradation products 1,2,4-triazole, triazole acetic acid, and triazole alanine, did not show any effect on AR nor on ER activity. This indicates that the triazole ring alone is not sufficient to exert effects on these receptors, but that side-groups are important too. Of the 9 tested triazines, we found deethyl-atrazine and ametryn to exert weak antiandrogenic activity. This is in line with other studies testing triazine herbicides, which have found either no or very limited binding of triazines to the AR and ER in competitive receptor binding assays (Tennant et al. 1994; Fang et al. 2003).

Regarding agonism and antagonism toward the ER, Kjærstad et al. (2010) reported antagonistic effects for propiconazole and tebuconazole which is in line with our findings. In the present study, all compounds that showed antagonism toward the AR also showed weak agonism or antagonism toward the ER. Similar findings where compounds affected both ER and AR activity have been reported for other groups of chemicals like benzophenones and brominated flame-retardants (Kawamura et al. 2005; Hamers et al. 2006). The tested PFAS showed no effects in the reporter gene assays, which is in line with one previous study using similar methods (Behr et al. 2018).

### Steroidogenesis

The gold standard to assess interactions with the steroidogenic pathway is the H295R assay, even though the in vivo relevance of the in vitro H295R assay is sometimes disputed (Tinwell et al. 2023). The H295R assay is described in the OECD test guideline 456 (OECD 2011), and prescribes to measure only the release of testosterone and estradiol. Our method allowed us to measure 13 steroid hormones simultaneously, which allows a much broader view of steroidogenic effects. In our H295R assay, exposure to the triazole fungicides and some of the triazines resulted in disrupted steroidogenesis. None of the tested PFAS showed any effect on steroidogenesis, which is in line with one previous study that also used H295R cells (Behr et al. 2018). Some of the triazoles were previously tested by Karmaus et al. (2016) with a different method using forskolin preinduced H295R cells in a 96-well format, but with results that are similar to ours. After hierarchical clustering analysis, 10 of the active compounds found in the present study clustered in 3 groups based on their steroidogenic profile. Two of the compounds that altered steroid hormone release, bitertanol and triadimenol, showed steroidogenic profiles distinct from the other compounds, and did not fit in any of the clusters.

Cluster I includes atrazine, deethyl-atrazine, and ametryn which resulted in increased levels of almost all measured steroid hormones. This effect has been described for atrazine before and is attributed to inhibition of phosphodiesterase-4, leading to increased cyclic adenosine monophosphate levels and increased activity of CYP450 enzymes (Sanderson et al. 2000; Kucka et al. 2012). Notably, atrazine is used as a reference compound for induction of steroidogenesis in the H295R assay (OECD 2011). In experiments with amphibians, exposure to atrazine and its metabolites, including deethyl-atrazine, induced structural gonadal abnormalities, and decreased fertility of both female and male animals (Sanderson 2006; Vandenberg et al. 2020). Similar effects were found in fish and rodents exposed to atrazine, increasing the evidence that atrazine is an EDC in vivo (Hayes et al. 2011). Our results suggest that ametryn, which is structurally similar to atrazine and shows the same steroidogenic profile, might induce similar effects.

**Fig. 6.**
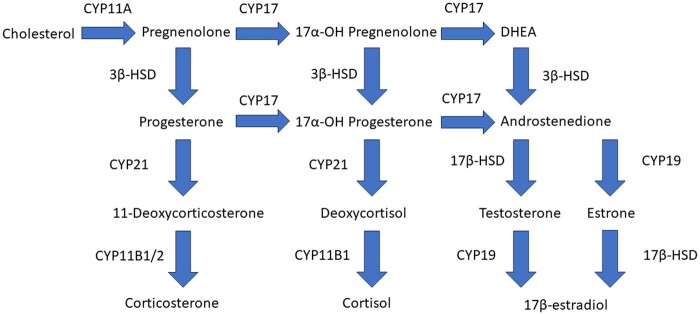
Steroidogenic pathway in H295R cells.

Cluster II includes tetraconazole, tebuconazole, cyproconazole, and paclobutrazol which led to up to 3.5-fold increased levels of progesterone and 17α-OH-progesterone compared with vehicle-treated controls, and decreased levels of androgens, estrogens, and corticosteroids. This effect of triazole fungicides has been described earlier and is caused by inhibition of CYP17 and CYP21 activity (Fig. 6) (Sanderson 2006; Karmaus et al. 2016). Prenatal and perinatal exposure to tebuconazole has been shown to cause feminization in male and virilization in female rat offspring, indicating decreased testosterone and estradiol levels (Taxvig et al. 2007). In the same study, increased concentrations of progesterone and 17α-OH-progesterone were found in the testis of male fetuses (Taxvig et al. 2007), which is in line with our findings. The weight-of-evidence for the causal relationship from disturbed steroidogenesis to developmental and reproductive effects is described in adverse outcome pathway AOP 288 from aopwiki.org (Society for Advancement of AOPs 2023). AOP 288 describes the relation between decreased CYP17 activity leading to decreased testosterone levels, consequently causing decreased AR activation leading to impaired testicular descent in mammals.

Cluster III includes fenbuconazole, difenoconazole, and propiconazole, which all increased levels of 17α-OH-progesterone more than 8-fold compared with levels in vehicle-treated controls, and decreased levels of corticosteroids, which indicates inhibition of CYP21 activity, but with a larger effect compared with cluster II. Exposure to fenbuconazole also decreased levels of androgens, which additionally indicates inhibition of CYP17 activity (Fig. 6). A recent review compiled extensive evidence of sex hormone disruption in many fish species after exposure to triazole fungicides (Huang et al. 2022). These effects include decreased circulating sex hormone concentrations after difenoconazole and propiconazole exposure. This is in line with our in vitro findings for propiconazole, but not difenoconazole.

Taken together, the in vitro effects in the compounds tested in the H295R steroidogenesis assay in the present study are in line with endocrine-disrupting effects found in vivo. Also, the measurement of 13 hormones instead of only testosterone and E2 gave us more mechanistic information on the affected enzymes. This allowed us to determine which compounds had similar mechanisms of action.

### Thyroid hormone system

Compounds that bind to TTR and thereby displace endogenous T4 can prevent the distribution of T4 to its target tissues (Ishihara et al. 2003). Lower levels of thyroid hormones in brain tissue can lead to decreased biological activity, leading to neurodevelopmental problems (Williams 2008). Furthermore, chemicals that have antagonistic effects on the TR can lead to similar decreases in the biological activity of thyroid hormones. Most PFAS and the triazoles difenoconazole, bitertanol, and 4methylbenzotriazole were active in our FITC-T4 TTR-binding assay. Two earlier studies in which the same PFAS were tested in the same assay found results similar to ours (Hamers et al. 2020; Langberg et al. 2023). Many studies have shown thyroid hormone system disruption by PFAS both in animal and epidemiological studies (Coperchini et al. 2021). Among other effects, PFAS were shown to induce hyperthyroidism, hypothyroidism, and alterations in TR gene expression in zebrafish, rats, and cats (Coperchini et al. 2021). To our knowledge, in vitro binding of the tested triazoles to human TTR has not been described before. Whereas propiconazole was known to be a TR antagonist in the GH3.TRE reporter gene assay (Paul Friedman et al. 2016) the TR-antagonistic effects of difenoconazole, triadimenol, and ametryn are also novel findings.

### In vitro hazard profiling

The results of the receptor activity assays, TTR-binding assay and H295R assay, were summarized to create in vitro hazard profiles of all 36 compounds (Fig. 5), allowing a rough comparison of the compounds based on their potency toward different molecular initiating events. Based on these profiles, the triazoles difenoconazole, bitertanol, fenbuconazole, tebuconazole and tetraconazole, and the triazine ametryn were concluded to be the most hazardous compounds, with a strong response in at least 2 readouts. The longer-chain PFAS, PFOS, PFOA, and PFHxS showed the highest potency in the TTR-binding assay. This could indicate that most attention should be placed on these compounds in the prioritization for further testing, environmental remediation, and prevention of pollution. In the case of PFOS, PFOA, and PFHxS this is already the case as those compounds have been phased out in the European Union.

The in vitro hazard profiles also show that the transformation products 1,2,4-triazole, triazole acetic acid, and triazole alanine showed no response in any of the assays. Triazole alanine and triazole acetic acid are plant metabolites, whereas 1,2,4-triazole is the most common metabolite in animals and soil (Sroher Kolberg et al. 2016). This strongly implies that metabolism should be considered when performing prioritization based on in vitro studies. This was taken into account by predicting metabolites through computational modeling and subsequently testing them for their bio-activity in vitro. This also holds true for persistent compounds like the ones we tested, as their environmental persistence does not necessarily cause them to be slowly metabolized in mammals. Still, the lack of response in our assays of triazole metabolites indicates that the metabolic degradation of triazole fungicides in polluted drinking water sources could be an effective way to decrease potential risks. However, additional studies are needed to confirm this also holds true for modes of action other than endocrine disruption.

The profiling performed in the present study is a first step to prioritize compounds based on toxicity for further testing, environmental remediation, and prevention of pollution. The next step in the prioritization strategy would be to come to a risk-based profiling, in which expected exposure concentrations are compared with the hazardous concentration. For example, in silico fugacity models can be used to estimate the concentrations of the compounds in drinking water (Cahill et al. 2003), which can then be compared with the hazardous concentrations provided by in vitro assays. Since in vitro assays do not account for the complexity of absorption, distribution, metabolism, and excretion in the human body, physiology-based kinetic (PBK) modeling can be performed to calculate drinking water concentrations into free internal concentrations. This free internal concentration can then directly be compared with the internal free benchmark concentration (BMC), which can be derived from the bioassay BMC (e.g., EC10 or EC50) by quantitative in vitro to in vivo extrapolation (QIVIVE) modeling. In the later stages of the ZeroPM project, all these steps will be taken to perform a risk-based prioritization of persistent and mobile compounds belonging to the triazole, triazine, and PFAS chemical classes.

## Supplementary Material

kfae131_Supplementary_Data
