# Supplementary material for: Profiling the endocrine-disrupting properties of triazines, triazoles, and short-chain PFAS
Source: Toxicol Sci. 2024 Oct 4;202(2):250–64. doi: 10.1093/toxsci/kfae131 (PMC11589101; doi:10.1093/toxsci/kfae131)
Supplement: kfae131_Supplementary_Data [file kfae131_supplementary_data.zip › kfae131_Supplementary_Data/toxsci-24-0254-File009.docx]

**Supporting information to**

**Endocrine disrupting effects of triazines, triazoles and short-chain PFAS.**

Maxim P. Carlier^1^, Peter H. Cenijn^1^, Timur Baygildiev^1^, Jenny Irwan^2^, Sylvia E. Escher^2^, Majorie B.M. van Duursen^1^, Timo Hamers^1^

1 Amsterdam Institute for Life and Environment, Section Environment and Health, Vrije Universiteit Amsterdam, 1081 HV, The Netherlands

2 Fraunhofer Institute for Toxicology and Experimental Medicine, Chemical Safety and Toxicology, 30625 Hannover, Germany

**Table S1: Internal standards and their supplier**

| **Internal standard** | **Supplier** |
| --- | --- |
| ^13^C3-17α-hydroxyprogesterone | Eurisotop |
| ^13^C2-Pregnenolone-2D_2_ | Eurisotop |
| ^13^C3-Testosterone | IsoSciences |
| Cortisol-D_4_ | Sigma-Aldrich |
| ^13^C3-Estrone (E1) | Sigma-Aldrich |
| ^13^C3-17β-estradiol | Sigma-Aldrich |
| 11-deoxycortisol- D_5_ | Sigma-Aldrich |
| DHEA-D_6_ | IsoSciences |
| ^13^C3-androstenedione | Sigma-Aldrich |
| Corticosterone-D_4_ | Sigma-Aldrich |
| ^13^C3-progesterone | Sigma-Aldrich |

**Table S2: IC50s (μM) with 95% Confidence Intervals of active compounds in the TTR-binding assay using the screening method and the initial method**

|  | Screening Method | | Initial Method | |
| --- | --- | --- | --- | --- |
|  | **IC50** | **95% CI of IC50** | **IC50** | **95% CI of IC50** |
| T4 | 7.2x10^-2^ | 5.9x10^-2^- 9.0x10^-2^ | 5.2x10^-2^ | 4.6x10^-2^- 5.9x10^-2^ |
| Bitertanol | 20.5 | 17.9- 23.4 | 62.7 | 36.1- 137 |
| Difenoconazole | 22.0 | 18.5- 26.5 | 38.5 | 28.6- 54.2 |
| 4-methylbenzotriazole | 26.3 | 22.8- 30.5 | 23.3 | 20.4- 26.9 |
| PFBS | 8.2 | 6.9- 9.8 | 8.3 | 7.6- 9.0 |
| PFHxS | 0.24 | 0.20- 0.30 | 0.27 | 0.25- 0.29 |
| PFOS | 0.19 | 0.16- 0.22 | 0.21 | 0.20- 0.22 |
| PFHxA | 2.5 | 2.1- 3.0 | 2.6 | 2.1- 3.2 |
| PFOA | 0.28 | 0.24- 0.31 | 0.28 | 0.27- 0.30 |
| GenX | 30.3 | 25.5- 36.4 | 31.9 | 26.8- 38.4 |


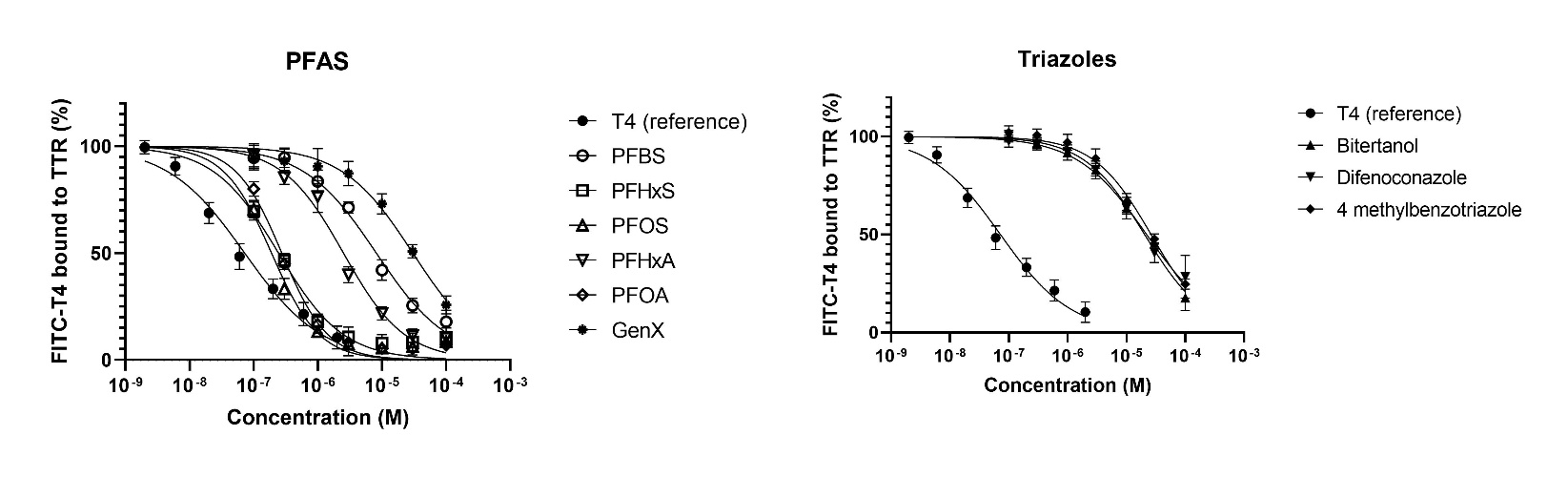


**Figure S1: Concentration-resonse curves of the active compounds in the TTR binding assay using the screening method. The concentration-response curves using the complete method are shown in figure 4.**

Principle component analysis (PCA) showed similar results to the hierarchical clustering analysis (Figure S2). The PCA score plot includes the tested compounds that showed statistically significant changes in one or more steroid hormone levels along two principal components (PCs) which explained 68.4% and 18.7% of the variation, respectively. The loading plot is superimposed on the score plot and shows the measured steroid hormones which are associated with the two PCs, i.e. the androgens, estrogens, corticosteroids, pregnenolone and 17α-OH-pregnenolone mostly with PC 1 and progesterone and 17α-OH-progesterone mostly with PC 2. The plot also shows that within PC 1, the corticosteroids group together below the x-axis and the androgens and estrogens group together above the x-axis. The PCA biplot shows tebuconazole, tetraconazole, cyproconazole and paclobutrazol clustering together in the lower right with decreased levels over all measured steroid hormones. Active triazines cluster together on the left side of the graph as they led to increased levels of androgens, estrogens and corticosteroids. Propiconazole, difenoconazole and fenbuconazole are clustered together in the upper part of the graph as exposure to these compounds resulted in the highest levels of progesterone and 17α-OH-progesterone.


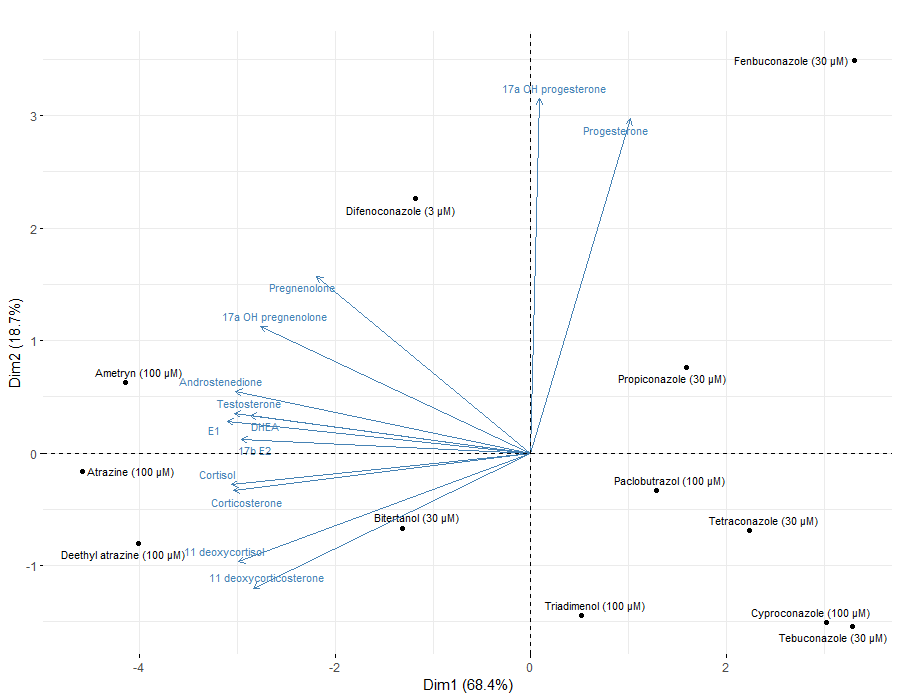


**Figure S2: PCA Biplot showing the loading plot containing the variables (steroid hormones) in blue with arrows and the score plot containing the tested compound (black dots) along two principal components (Dim1 and Dim2). The concentration in parentheses is the highest, non-cytotoxic concentration of each compound.**
